# L-dopa-Dependent Effects of GLP-1R Agonists on the Survival of Dopaminergic Cells Transplanted into a Rat Model of Parkinson Disease

**DOI:** 10.3390/ijms222212346

**Published:** 2021-11-16

**Authors:** Osama F. Elabi, Jeffrey S. Davies, Emma L. Lane

**Affiliations:** 1 School of Pharmacy and Pharmaceutical Sciences, Cardiff University, Cardiff CF10 3NB, UK; 2 Institute of Life Sciences, School of Medicine, Swansea University, Swansea SA2 8PP, UK; jeff.s.davies@swansea.ac.uk

**Keywords:** exendin-4, liraglutide, L-dopa, cell transplantation, neuroprotection, Parkinson Disease

## Abstract

Cell therapy is a promising treatment for Parkinson’s disease (PD), however clinical trials to date have shown relatively low survival and significant patient-to-patient variability. Glucagon Like Peptide-1 receptor (GLP-1R) agonists have potential neuroprotective effects on endogenous dopaminergic neurons. This study explores whether these agents could similarly support the growth and survival of newly transplanted neurons. 6-OHDA lesioned Sprague Dawley rats received intra-striatal grafts of dopaminergic ventral mesencephalic cells from embryonic day 14 Wistar rat embryos. Transplanted rats then received either saline or L-dopa (12 mg/kg) administered every 48 h prior to, and following cell transplantation. Peripheral GLP-1R agonist administration (exendin-4, 0.5 μg/kg twice daily or liraglutide, 100 μg/kg once daily) commenced immediately after cell transplantation and was maintained throughout the study. Graft survival increased under administration of exendin-4, with motor function improving significantly following treatment with both exendin-4 and liraglutide. However, this effect was not observed in rats administered with L-dopa. In contrast, L-dopa treatment with liraglutide increased graft volume, with parallel increases in motor function. However, this improvement was accompanied by an increase in leukocyte infiltration around the graft. The co-administration of L-dopa and exendin-4 also led to indicators of insulin resistance not seen with liraglutide, which may underpin the differential effects observed between the two GLP1-R agonists. Overall, there may be some benefit to the supplementation of grafted patients with GLP-1R agonists but the potential interaction with other pharmacological treatments needs to be considered in more depth.

## 1. Introduction

Parkinson’s disease is the second most common neurodegenerative disease affecting 6.9 million people around the world [1]. Clinically, PD is characterized by rigidity, bradykinesia, and resting tremor alongside a range of non-motor symptoms [2]. The main pathological feature of the disease is the progressive degeneration of the nigrostriatal dopaminergic neurons resulting in depletion of striatal dopamine. Currently, there are no disease modifying treatments, with all available therapies providing symptomatic relief alone. As the disease progresses, these treatments have progressively limited efficacy, simultaneously causing greater levels of side effects and adversely affecting the patient’s quality of life. One of the potential therapeutic approaches that can achieve long-lasting relief for the motor and non-motor symptoms is cell replacement therapy [3,4,5]. This approach is based on restoring the lost striatal dopamine by transplanting dopaminergic neurons ectopically into the striatum. Originally, proof of principle studies utilised primary cells from embryonic ventral mesencephalon. Randomised controlled trials demonstrated functional benefit in some patients but lacked consistency, leading to highly variable outcomes and bringing to the forefront motor side effects, now known as graft-induced dyskinesia [6,7]. The most recent clinical trial, TransEUro [8], took place with a more refined tissue preparation and patient selection protocol with a view to improving these outcomes ahead of upcoming clinical trials with dopaminergic neuronal precursors derived from human embryonic stem cell or induced pluripotent stem cell lines [9,10].

One of the biggest challenges in cell transplantation, shown in both clinical and pre-clinical trials, is the low apparent survival rate of transplanted cells and in particular the dopaminergic neurons [11,12] reviewed in [13]. Critical to functional recovery however is not only sufficient graft size but also good fibre innervation to sufficiently and evenly innervate the dopamine depleted striatum [14]. The transplanted dopaminergic neurons are vulnerable to many factors which contribute to the early cell death, including oxidative stress, low trophic factors, excitotoxicity, and inflammatory cytokines [13,15,16]. The largest challenge to the cells is likely to be in the early stages following cell transplantation, and several neuroprotective agents have been explored to promote survival including neurotrophic factors, anti-apoptotic agents, and anti-oxidants [14,17] (reviewed in [13]). Glucagon-like peptide-1 receptor agonists (GLP-1R) are employed in the treatment of type 2 diabetes [18,19] but have recently been of interest in their potential to modify the progression of a range of neurological disorders [20,21] including PD [22,23]. Convincing preclinical data [22,24,25] led to a randomised controlled trial of the first GLP-1R agonist to be used in the treatment of diabetes, exendin-4 which showed a mild but significant beneficial effect on the motor scores of people with PD [26]. Many preclinical studies have now also illustrated the effectiveness of other GLP-1R agonists to protect nigral dopaminergic neurons from degeneration, and thus support maintenance of motor function in a range of animal models of PD [27]. Furthermore, exendin-4 has been shown to promote neurogenesis [28] and produce both neurotrophic and antiapoptotic actions [29,30] features which could promote survival of transplanted cells. In addition to the benefit of being established therapeutic agents with a good safety record, exendin-4 and liraglutide have the advantage of readily crossing the blood-brain barrier with a substantial half-life, making them practical for clinical use in this context.

**Figure 1:**
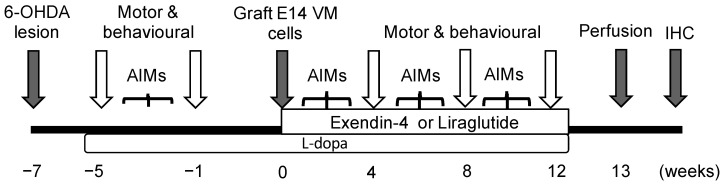
Schematic illustration for experiment time line: 6-OHDA lesion induced at week 7 before cell transplantation; L-dopa treatment started at week 5 before cell transplantation and continued for 12 weeks post transplantation. GLP-1R agonist therapy started immediately after cell transplantation and continued for 12 weeks. Motor tests and AIMs were recorded at intervals prior to, and following cell transplantation. All rats were perfused 13 weeks post-transplantation. AIMs = abnormal involuntary movements.

We hypothesized that both exendin-4 and liraglutide could support the survival and efficacy of allogenic ventral mesencephalon VM cell transplantation in the 6-OHDA rat model. We have tested this hypothesis in a clinically relevant animal model by using allograft transplantation to simulate a level of graft-host immunological reaction not observed with syngeneic grafts, and adding exposure to chronic L-dopa, before and after cell transplantation to simulate a patient candidate for transplantation (see Figure 1).

## 2. Results

Animals were weighed regularly to monitor the effects of the drugs. Rats treated with L-dopa showed a statistically significant but small (3.5%) increase in weight not seen in the liraglutide and exendin-4 treated groups (data not shown). Weights were otherwise consistent between all treatment groups. Post-mortem histological analysis confirmed an almost complete lesion of the dopaminergic neurons of the substantia nigra in all animals; the mean percentage TH^+^ cell loss on the lesioned nigra was greater than 97% of the intact nuclei in each experimental group (Supplementary Figure S1). It was then important to determine in this paradigm that GLP-1R agonists had no direct effects on the nigrostriatal pathway or motor function. Exendin-4 did not affect TH^+^ cell counts in the nigra nor evoke any functional improvement in amphetamine-induced rotations, vibrissae, or adjusting paw step test, and performance worsened in the cylinder test (Supplementary Figure S2A–D).

To assess the capacity of the E14 VM graft to reverse motor deficit in the lesioned rats, we evaluated the motor tests between the lesion-only group and E14 VM cell graft group using amphetamine-induced rotation, vibrissae, stepping, and cylinder tests. As expected, the graft significantly reduced the number of ipsilateral rotations in the amphetamine-induced rotation test and significantly improved performance in the cylinder test at week 12 (unpaired *t*-test: amphetamine test, *p* < 0.0001; cylinder test *p* < 0.001). However, the graft had no effect on performance in the vibrissae and stepping tests vibrissae test (Supplementary Figure S2E–H). This finding indicates that the graft in this instance was capable of partially alleviating the motor dysfunction induced by the lesion in this model, but was not of sufficient size to produce complete functional recovery.

### 2.1. GLP-1R Receptor Expressed on the E14 VM Cells and Persists Post-Transplantation

**Figure 2:**
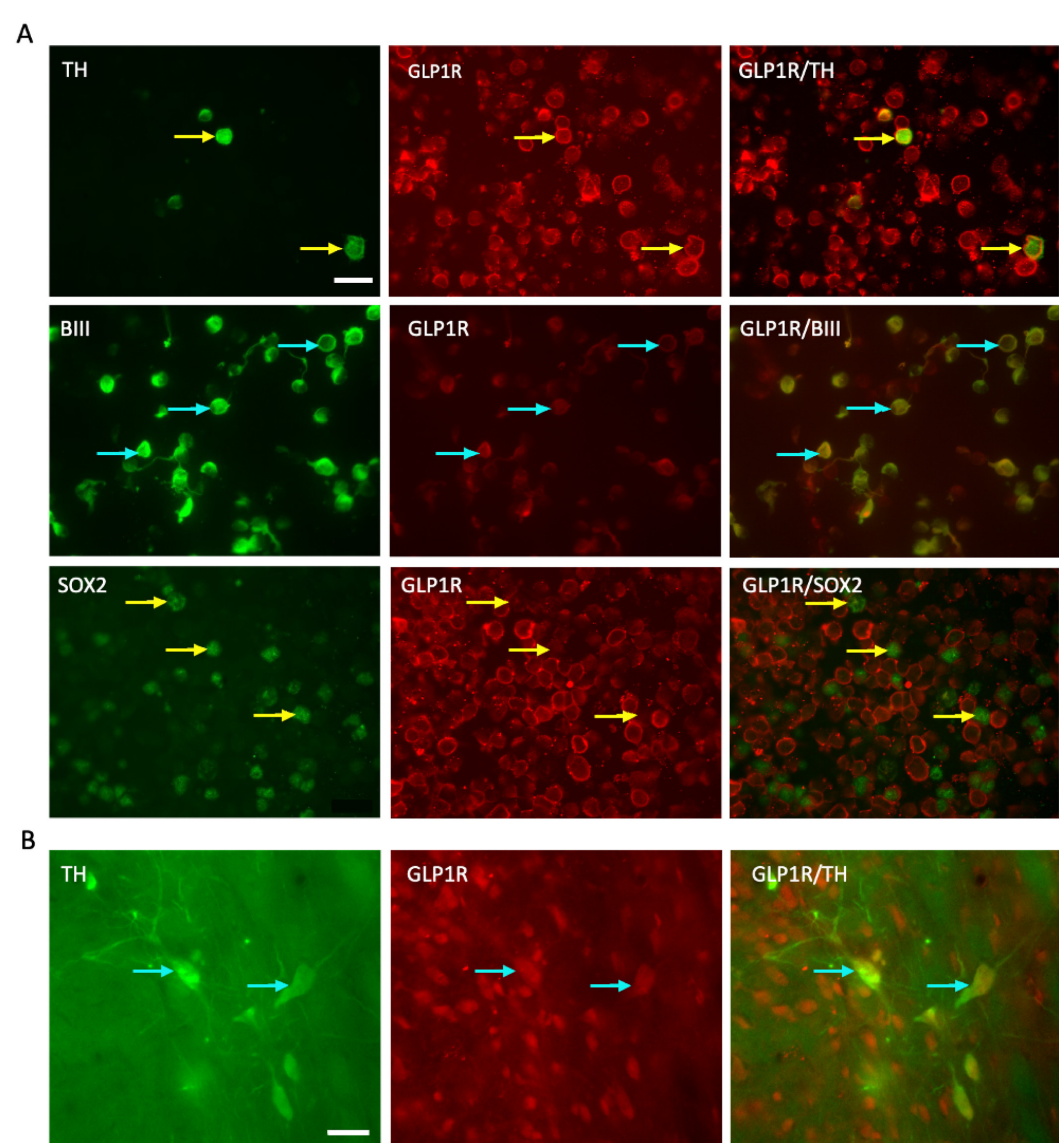
GLP-1R expression on the E14 VM cells and dopaminergic neuronal graft in the striatum. (**A**) florescent images showing co-expression of GLP-1R (red) with TH+ cells (green), BIII tubulin+ cells (green) and SOX2+ cells (green) in E14 VM cells before grafting. (**B**) florescent images showing GLP-1R expression with TH+ cells in the grafted striatum after 13 weeks of transplantation (The arrow points to the co-localised points). Scale bar (**A**,**B**) = 20 μm. TH = tyrosine hydroxylase; GLP-1R = glucagon like peptide -1 receptor.

The ability of GLP-1R agonists to produce a direct effect on the grafted cells requires GLP-1R in grafted cells. We examined the expression of the GLP-1R within E14 VM cell populations including BIII tubulin^+^ neurons, TH^+^ dopaminergic neurons, and SOX2^+^ neural stem cells. GLP-1R was expressed on the mature neurons and importantly, on the dopaminergic neurons, but not expressed on stem-like, SOX2^+^ cells (Figure 2A). Western blot confirmed expression of GLP-1R protein in E14 VM, as well as different brain regions of adult female Sprague Dawley rats including the frontal cortex, area including the substantia nigra, hippocampus, and striatum (Supplementary Figure S3). Histological analysis of grafts after 12 weeks showed co-expression of the GLP-1R with TH^+^ cells indicating the persistence of the receptor in grafted cells throughout the experimental period (Figure 2B). This finding confirmed the existence of the main machinery for the GLP-1R agonists to produce an effect on the grafted cells and host environment.

### 2.2. Differential Effects of Exendin-4 and Liraglutide on Graft Function in the Presence of L-dopa

**Figure 3:**
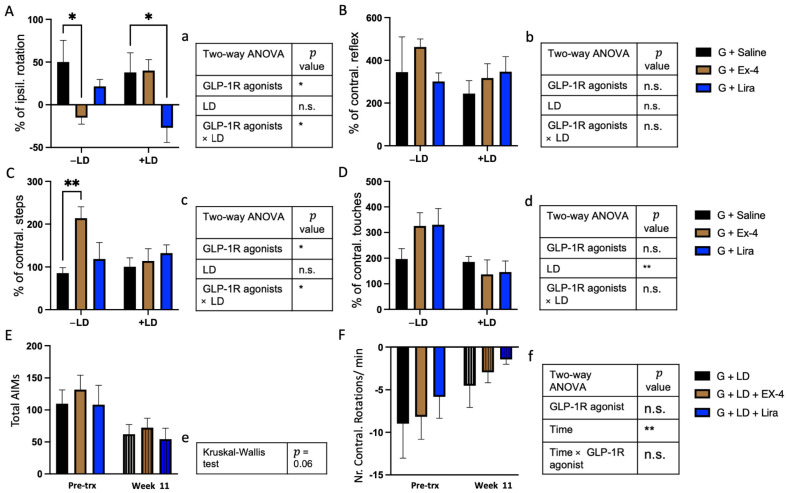
The effect of GLP-1R agonists and L-dopa on the motor and L-dopa induced dyskinesia tests. (**A**) Amphetamine induced rotation test, (**B**) vibrasae test, (**C**) stepping test and (**D**) cylinder test on week 12 post transplantion of rats received either saline, Ex-4 or Lira in −/+ LD groups. The data were expressed as a percentage of the baseline (pre-transplantation time point) in number of ipsilateral rotations, contralateral reflex, contralateral steps and contralateral touches, respectively. (**E**) AIMs (**F**) L-dopa induced rotation received either Ex-4, Lira or no GLP-1R agonists in +LD treated groups at pre-trx and week 11 time points. The data were expressed as total number of AIMs or number of contralateral rotations per min, respectively. (**a**–**f**) illustration of statistical summary of (**A**–**F**) respectively. (**A**–**D**,**F**) two-way ANOVA, (**E**) Kruskal-Wallis test, * *p* < 0.05, ** *p* < 0.01. *n* = G − LD (7), G + LD (8), G − LD + Ex-4 (9), G + LD + Ex-4 (8), G − LD + Lira (6), G + LD + Lira (7). n.s. = not significant, LD = L-dopa; Ex-4 = Exendin-4; Lira = Liraglutide; Pre-trx = Pre- transplantation.

We examined whether exendin-4 and liraglutide could impact graft function using amphetamine-induced rotation, vibrissae, stepping, and cylinder tests, 12 weeks post-transplantation both with and without concomitant L-dopa. In the absence of L-dopa, GLP-1R agonists improved performance in the amphetamine-induced rotation, and stepping tests but not in the cylinder, and vibrissae tests [two-way ANOVA: amphetamine test (GLP-1R agonists, F_(2,37)_ = 3.9, * *p* < 0.05, LD, F_(1,37)_ = 0.1, n.s., GLP-1R agonists × LD, F_(2,37)_ = 4.87 * *p* < 0.05); vibrissae test (GLP-1R agonists, F_(2,39)_ = 0.2, n.s., LD, F_(1,39)_ = 1 n.s., GLP-1R agonists × LD, F_(2,39)_ = 1.4, n.s.) stepping test (GLP-1R agonists, F_(2,39)_ = 4.35, * *p* < 0.05, LD, F_(1,39)_ = 1.33, n.s., GLP-1R agonists × LD, F_(2,39)_ = 3.7, * *p* < 0.05); cylinder test (GLP-1R agonists, F_(2,38)_ = 0.54, n.s., LD, F_(2,38)_ = 10.8, ** *p* < 0.01, GLP-1R agonists x LD, F_(2,38)_ = 2.3, n.s.)] (Figure 3A–D). However, pairwise comparisons showed that exendin-4 improved graft function only in the non-L-dopa (−LD) group, with no effect in L-dopa (+LD) treated groups (Figure 3A–D).

Liraglutide had no effect on motor behaviours in the presence of the graft alone (−LD) but improved graft function in the +LD group in the amphetamine-induced rotation test (Figure 3A–D).

Grafts have previously been shown to alleviate the LID (L-dopa induced dyskinesia) induced by L-dopa [31,32,33], evaluated in this study using the AIMs score and L-dopa-induced rotations (Figure 3E,F). The analysis of total AIMs score and L-dopa induced contralateral rotations at week 11 post-transplantation showed that whilst AIMS reduced over time (although not significant, Kruskal-Wallis test, *p* = 0.06), there was no apparent difference between the three grafted groups treated with LD. Importantly there was a significant reduction of L-dopa induced contralateral rotations at week 11 compare to pre-transplantation (two-way ANOVA: GLP-1R agonists, F_(2,20)_ = 0.32 n.s., time, F_(1,20)_ = 9.7, ** *p* < 0.01, GLP-1R agonists × time, F_(2,20)_ = 0.04, n.s.) (Figure 3E,F).

### 2.3. GLP-1R Agonists Induced Alterations in the Histological Characteristics of the Graft

**Figure 4:**
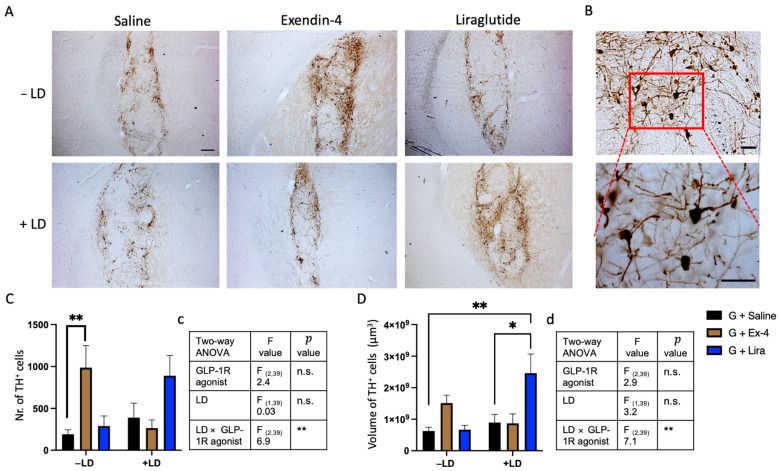
The effect of GLP-1R agonists and L-dopa on the number and volume of the graft TH+ cells: (**A**) bright field images captured at 4× magnification showing TH+ graft in the striatum of the rats received either saline, exendin-4 or liraglutide and treated +/− LD. (**B**) bright field image of the TH+ cell captured at 10× magnification (upper) and 40× magnification (lower) from the graft in the striatum of a rat in Ex-4/−LD group showing the TH^+^ cell morphology. (**C**,**D**) the TH^+^ cell counting and the volume of TH^+^ cells respectively in the graft of the rats received either saline, exendin-4 or liraglutide and treated +/− LD. (**c**,**d**) illustration of statistical sumary of (**C**,**D**) analysis respectively. Two way ANOVA * *p* < 0.05, ** *p* < 0.01. *n* = G − LD (7), G + LD (8), G − LD + Ex-4 (9), G + LD + Ex-4 (8), G − LD + Lira (6), G + LD + Lira (7). n.s. = not significant LD = L-dopa; Ex-4 = Exendin-4; Lira = Liraglutide TH = tyrosine hydroxylase. Scale bar = (**A**) 200 μm, (**B**) 50 μm.

To assess the impact of the GLP-1R on protecting the transplanted dopaminergic neurons and their distribution in the striatum, we counted the number of surviving TH^+^ cells and volume of the TH^+^ graft in the striatum (Figure 4A,C,D). Whilst there was no main effect of GLP-1R agonist or L-dopa treatment, there was a significant interaction between the two factors [two-way ANOVA: TH^+^ cells count (GLP-1R agonists, F_(2,39)_ = 2.4, n.s, LD, F_(1,39)_ = 0.03, n.s., GLP-1R agonists × LD, F_(2,39)_ = 6.9, ** *p* < 0.01); TH^+^ volume (GLP-1R agonists, F_(2,39)_ = 2.9, n.s., LD, F_(1,39)_ = 3.2, n.s., GLP-1R agonists × LD, F_(2,39)_ = 7.1, ** *p* < 0.01)]. Pairwise comparisons confirmed increased TH^+^ cell count in the exendin-4 treated, −LD group compared to saline, which was not observed in the +LD group. There was a small but non-significant increase in graft volume also detected. Conversely, liraglutide alone had no effect on the TH^+^ cell content of the graft but in the presence of L-dopa there was an increase which failed to reach significance (*p* = 0.103) which was paralleled by a significant increase in graft volume compared to saline-treated groups.

### 2.4. L-dopa and GLP-1R Agonists Altered the Inflammatory Response around the Graft

**Figure 5:**
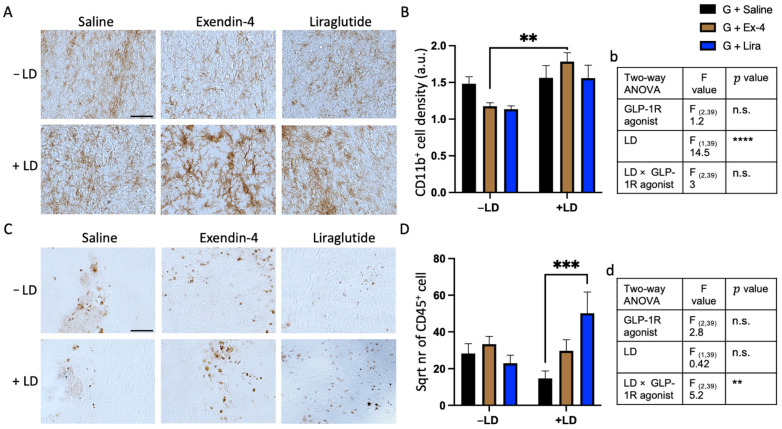
Quantification of CD11b+ microglia and CD45+ leukocyte in the striatum of the grafted rats. (**A**,**B**) Bright field images showing CD11b+ cells density and morphology around the graft and their quantification respectively in the striatum of the rats received either saline, exendin-4 or liraglutide and +/− LD. (**C**,**D**) Bright field images showing CD45+ cells number and their quantification respectively in the striatum of the rats received either saline, exendin-4 or liraglutide and +/− L-dopa. (**b**,**d**) illustration of statistical sumary of (**B**,**D**) analysis respectively. Two way ANOVA ** *p* < 0.01, *** *p* < 0.001, **** *p* < 0.0001. *n* = G − LD (7), G + LD (8), G − LD + Ex-4 (9), G + LD + Ex-4 (8), G − LD + Lira (6), G + LD + Lira (7). n.s. = not significant LD = L-dopa; Ex-4 = Exendin-4; Lira = Liraglutide TH = tyrosine hydroxylase. Scale bar (**A**,**C**) = 100 μm.

We next investigated whether the GLP-1R agonists −/+ LD had an effect on the density of CD11b^+^ microglia and/or CD45^+^ leukocyte infiltration in the grafted area. For CD11b^+^ cell analysis, L-dopa significantly increased microglial density around the graft, which was particularly evident in the exendin-4 treated group (two-way ANOVA: CD11b^+^ optical density (GLP-1R agonists, F_(2,39)_ = 1.2, n.s, LD, F_(1,39)_ = 14.5, **** *p* < 0.0001, GLP-1R agonists × LD, F_(2,39)_ = 3, n.s.). (Figure 5A,B). For CD45^+^ cells, there was no main effect of L-dopa or GLP-1R agonist on the number of leukocyte cells in the grafted striatum but a significant interaction between the two treatments (two-way ANOVA: CD45^+^ cells count (GLP-1R agonists, F_(2,39)_ = 2.8, n.s, LD, F_(1,39)_ = 0.42, n.s., GLP-1R agonists × LD, F_(2,39)_ = 5.2, ** *p* < 0.01). The pairwise comparisons howed that in the absence of L-dopa liraglutide had no effect, but in the + LD group significantly increased CD45^+^ cells compared to the graft/+ LD group. Exendin-4 had no effect in the presence or absence of L-dopa (Figure 5C,D).

### 2.5. Exendin-4 Paradoxically Caused Insulin Resistance in the Grafted Rats Treated with L-dopa

**Figure 6:**
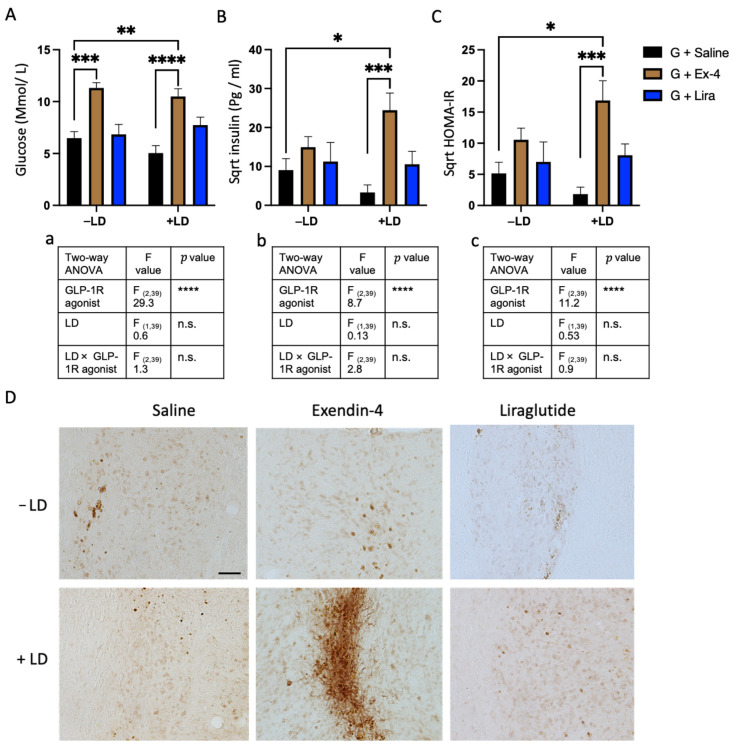
Evaluation of insulin resistance in the plasma in the graft of the transplanted rats: (**A**) glucose level, (**B**) insulin level, (**C**) HOMA-IR value of the transplanted rats received either saline, exendin-4 or liraglutide and +/− LD. (**a**–**c**) illustration of statistical sumary of (**A**–**C** analysis respectively. (**D**) bright field images showing phosphorylated insulin receptors labelled with IRS-1 pS1011 in and around the graft of rats received either saline, exendin-4 or liraglutide and +/− LD. Two way ANOVA * *p* < 0.05, ** *p* < 0.01, *** *p* < 0.001, **** *p* < 0.0001. *n* = G − LD (7), G + LD (8), G − LD + Ex-4 (9), G + LD + Ex-4 (8), G − LD + Lira (6), G + LD + Lira (7). n.s. = not significant; LD = L-dopa; Ex-4 = Exendin-4; Lira = Liraglutide. Scale bar = 200 μm.

We next investigated whether GLP-1R agonists and L-dopa had an effect on the fasting plasma level of insulin, glucose, and the insulin resistance index (HOMA-IR) values. GLP-1R agonists had a significant overall effect, increasing glucose, insulin, and HOMA-IR values [two-way ANOVA: glucose level, insulin level, HOMA-IR value (glucose: GLP-1R agonists, F_(2,39)_ = 29.3, **** *p* < 0.0001, LD, F_(1,39)_ = 0.6, n.s., GLP-1R agonists × LD, F_(2,39)_ = 1.3, n.s) (insulin: GLP-1R agonists, F_(2,39)_ = 8.7, **** *p* < 0.0001, LD, F_(1,39)_ = 0.13, n.s., GLP-1R agonists × LD, F_(2,39)_ = 2.8, n.s) (HOMA-IR: GLP-1R agonists, F_(2,39)_ = 11.2, **** *p* < 0.0001, LD, F_(1,39)_ = 0.35, n.s., GLP-1R agonists × LD, F_(2,39)_ = 0.9, n.s)]. The pairwise comparison between the groups showed that exendin-4 significantly increased glucose in the −/+ LD groups compared to the grafted groups and it increased the insulin and HOMA-IR values only in the +LD group compared to the graft control group +/− LD (Figure 6A–C). For liraglutide, the pairwise comparison showed that it had no effect in the +/− LD rats suggesting that the changes in plasma glucose and insulin were associated with exendin-4 only.

Next, we explored whether insulin resistance had developed in the grafted cells. We used a phosphorylated insulin receptor marker (IRS-1 pS1011) to stain the graft area. The images showed that the graft area of the exendin-4 (Ex-4)/+LD group has a highly distinctive level of IRS-1 pS1011 with neuronal cytoplasmic shape, while it has a lower density with a rounded nuclear shape in all other transplanted groups (Figure 6D). This morphology is indicative of the development of insulin resistance in the graft of the Ex-4/+LD group [34].

## 3. Discussion

In this study, we describe for the first time the effects of GLP1-R agonists on primary cell transplants in a rodent model of PD. We confirmed the presence of GLP-1R on VM dopaminergic neurons (E14) at the time of transplantation suggesting the availability of the mechanical components for the GLP-1R agonist to produce an effect on graft survival confirming previous findings [35,36,37]. Post-mortem analysis 13 weeks after transplantation also confirmed the persistence of this receptor on the mature transplanted dopaminergic neurons. Both GLP-1R agonists appear able to support the survival and function of the transplanted cells, but there was a differential interaction between these compounds and L-dopa. This manifested in contrasting effects, that exendin-4 support of graft survival was lost in rats treated concurrently with L-dopa, whilst liraglutide only produce a supporting effect in the presence of L-dopa. Unexpectedly, we found co-administration of exendin-4 and L-dopa induced an insulin resistance phenotype which was not observed with liraglutide.

To contextualise our data with previous studies, we did not identify any effects of the GLP-1 agonists on the nigrostriatal pathway of control or transplanted animals. This is unsurprising given the delayed timing of the administration of the GLP-1 agonists following administration of 6-OHDA. Early studies, showing the ability of exendin-4 to promote recovery of the nigrostriatal pathway in this model, started exendin-4 treatment 7 days after the lesion was initiated [24,38]. Nevertheless, it is important to reiterate that neuroprotective effects can only manifest if there are cells there to support, highlighting the clinical need for biomarkers for confident early detection of Parkinson’s disease to enable earlier intervention [39]. We show here that these effects are extended to exogenously implanted dopaminergic cells. Exendin-4 substantially improved graft survival with a graft cell number in excess of six times that of the grafted animals alone, competitive with similar studies that seek to improve graft survival through different approaches [14,40,41,42].

Liraglutide has been less consistently found to be neuroprotective, as some studies failed to show any effect of this particular GLP-1 agonist on the substantia nigra in the 6-OHDA model, but benefits have been shown when rotenone or MPTP were used as the dopamine depleting agent [43,44,45]. This difference has been ascribed to a reduced ability of liraglutide to enhance GDNF release and inhibit mitochondrial mitophagy signaling [29]. Similarly, we have shown here no effect of liraglutide on transplanted cells when given alone. In the current study the overall survival of transplanted cells was relatively small, mirrored by the low functional recovery observed in the behavioural analysis [46,47]. Nevertheless, the grafts were functional and able to reduce asymmetric rotations following amphetamine administration, indicating adequate release of dopamine to counterbalance the dopamine levels on the intact side.

The inclusion of L-dopa is supported by 2 rationales; L-dopa is a first line therapy for Parkinson’s disease and many patients will be taking it at the time of transplantation should this be an emerging treatment. Second, L-dopa triggers LID, a treatment limiting motor side effect with few effective management strategies. In considering the first of these, the effects of L-dopa alone on the graft in this study (increasing the level of microglia) are consistent with a previous study in which L-dopa had no direct effect on the survival of a VM primary tissue transplant but it increased the host microglial response to a xenograft [48]. Microglia are thought to play a key role in inflammation in PD and in the generation of LID [49], whilst insulin signaling may alter microglial activation in concentration-dependent manner [50]. Furthermore, microglial have been identified as a potential source of GLP-1 which are affected by inflammatory stimuli [51]. This complex interaction needs to be explored in more depth but a transplant model which requires histological analysis may not be the most suited. L-dopa has been shown to contribute to increased infiltration of CD45^+^ cells around the xenograft and prompt infiltration of CD4^+^ (T_h_ lymphocytes) rather than CD8^+^ (cytotoxic T lymphocyte) cells [48], suggesting that this occurs more readily in less immune compatible grafts than the allografting model use here. Why exendin-4 and liraglutide differentially affect graft survival in the presence of L-dopa is unresolved but there were differences in the immune response and the peripheral response of glucose that could provide indicators. The paradoxical development of insulin resistance in exendin-4, L-dopa treated rats could be the reason for L-dopa preventing exendin-4 from supporting graft survival and function. Insulin resistance has been shown to cause microvascular alterations, pericyte depletion, and changes in microglial distribution pointing to aggravation in the vascular pathology in the striatum of the 6-OHDA lesioned mouse [52]. This indicates that insulin resistance causes changes in the micro-environment of the host striatum that could lead to effects on graft survival. Another study showed that the nigral dopaminergic neurons are more vulnerable to degeneration when exposed to the toxin MPTP in a mouse model of type 2 diabetes mellitus [53,54] consistent with many clinical studies suggesting aggravation of PD with existing comorbidity of diabetes [55,56,57]. The elevation of microglial density around the graft of the exendin-4 group/ + LD compared to exendin-4/-LD lends credence to this hypothesis, and accumulation of activated microglia around the graft has been shown to limit graft survival and function [7,58,59]. Peripheral insulin resistance has been linked with the release of pro-inflammatory cytokines which can cross the blood-brain barrier (reviewed in [60]), by increasing the inflammatory reaction around the graft the supportive effect of exendin-4 on graft survival may be prevented. So, it is plausible that developing insulin resistance potentiates several factors leading to the promotion of death of the transplanted cells or an impairment in their function.

In contrast to exendin-4, no evidence of insulin resistance was observed in the presence of liraglutide, although it is also noteworthy that they expected increase in fasting glucose observed with exendin-4 was not observed. This could indicate reduced efficacy of liraglutide in this model and that higher doses might be required to see similar effects. However, the enhanced graft survival and volume was only evident when the combination of of liraglutide and L-dopa were administered. This could be the result of two potential synergistic mechanisms; an anti-inflammatory response or effects on GDNF. In combination with L-dopa the infiltration of CD45^+^ cells increased around the graft. L-dopa induces differentiation of T_h_2 over T_h_1 subtypes, likely stimulating the release of anti-inflammatory cytokines [61,62]. Interestingly, GLP-1R agonists have shown the ability to polarise macrophages from M1 (pro-inflammatory) to M2 (anti-inflammatory) via stimulation of the STAT-3 pathway. In addition, it significantly increased the anti-inflammatory cytokines like IL-10 [63]. So, L-dopa and liraglutide possibly modified the inflammatory reaction around the graft to support graft function which was not potent enough when delivered alone. However, further investigation is needed to characterize the type of inflammatory cells around the graft. Both L-dopa and GLP-1 agonists have also been shown to raise GDNF levels; it is possible at the doses given that neither were able to induce sufficient increase when administered alone but a synergistic effect could result in the observed increase in survival.

While some preclinical studies report a protective effect of exendin-4 on the generation of LID, this can largely be ascribed to the neuroprotective effects at the nigra [24,64]. Hypersensitivity of the striatal output pathways leading to LID is only generated with substantial nigral degeneration and if this is negated by a neuroprotective agent it would be expected that a lower level of LID would be induced by L-dopa. Exceptionally, Yu and colleagues [65] showed that an extended release form of exendin-4, PT320, did reduce LID severity in the presence of a full, stable nigrostriatal lesion. Direct effects on LID were not the focus of this study but there is no evidence to suggest that over the 12 week study period that exendin-4 or liraglutide had a direct independent effect on LID, the trajectory of reduction in LID being identical to that of the grafted only animals suggesting that the graft alone is responsible for any changes in L-dopa response. The difference between these studies may be due to the different formulation and thus half life, of exendin-4, but it is worthy of further enquiry.

The 6-OHDA lesioned rat does not replicate all the features of Parkinson’s disease, and the neuroprotective effects of GLP-1R agonists may not be as evident in α-synuclein based models of PD. Some studies show a neuroprotective effect similar to that shown in the 6-OHDA lesioned rat [29,66,67] whilst others illustrate an increase in α-synuclein accumulation which could promote its toxic effects in some regions of the brain [68]. Post mortem studies have demonstrated a relatively slow accumulation of α-synuclein in grafted cells [69,70] and whilst this is not currently thought to lead to major issues with graft function, it would be prudent to verify effectiveness of GLP-1R agonists in this scenario. Both a potential role of GLP-1 agonists in α-synuclein accumulation and the differential effects in the presence of L-dopa serve to highlight important considerations in the development of translational models to evaluate putative therapeutic interventions. Clinical trials in L-dopa naïve patients are rare and challenging, given that the majority of patients require some form of dopaminergic therapy within a year of treatment to enable them to maintain quality of life [6,7]. Nevertheless, few preclinical studies seek to evaluate their putative therapeutic or protective agents in a model that includes L-dopa treatment. Specifically for transplantation, whilst patients will likely be taking L-dopa at the time of transplantation and will continue to do so until they show improvement in motor function, few studies have explored the impacts of putative therapeutic agents in the presence of the most widely used drug for the management of PD. Here we show two agents, with very similar pharmacology, being differentially impacted by the presence of L-dopa.

## 4. Material and Methods

### 4.1. Animals

Adult Sprague Dawley rats and time mated pregnant Wistar rats were obtained from Envigo. They kept in animal house at 20 °C temperature e, 45% humidity and 12 h light cycle. They supplied with dl libitum access to food (14% protein, Harlan) and water. The experiment conducted in agreement with the Animals Scientific Procedure Act 1996 and Home Office regulation.

### 4.2. Experimental Design

A complete unilateral degeneration of the nigral dopaminergic neurons was established by infusion of 6-hydroxydopamine (6-OHDA) unilaterally into the medial forebrain bundle MFB of 90 Sprague Dawley rats. Motor tests including amphetamine rotation, stepping, vibrissae and cylinder tests were evaluated two weeks post lesion. In the amphetamine rotation test, the rats who had ipsilateral rotations more than 6 per minute were considered to have degeneration more than 90% of the dopaminergic neurons [71]. The animals were then separated into two sets: set 1 includes two balanced groups, either received exndin-4 (*n* = 8) or saline (*n* = 8); set 2 includes one group received saline only (*n* = 9) and six groups transplanted with Wister e14 VM cells intrastriatally. The grafted groups (G) were subdivided depending on their treatments: 3 groups received L-dopa (+LD), 3 received saline (−LD). One group from each was also treated with either exendin-4 (Ex-4) or liraglutide (Lira) [G + LD (*n* = 8) G + LD + Ex-4 (*n* = 8), G + LD + Lira (*n* = 7), *n* = G − LD (*n* = 7) G − LD + Ex-4 (*n* = 9), G − LD + Lira (*n* = 6)]. The cell transplantation surgery was performed after priming the lesioned rats with L-dopa treatment for 5 weeks. The motor tests were carried out at different time points: before starting the L-dopa, prior to cell transplantation and at weeks 4, 8 and 12 post transplantation. LID were evaluated by recording Abnormal Involuntary Movements (AIMs) and L-dopa-induced rotations at intervals (Figure 6). 12 rats were excluded from the study due to incomplete lesion and 8 rats were excluded due to unsuccessful transplantation (no transplanted cells detected in the graft area).

### 4.3. Surgical Procedure

All the surgeries were performed using aseptic techniques with a Kopf stereotactic frame and a Harvard micro-drive infusion pump. The rats were anaesthetised with isoflurane 5% at induction and 2–3% at maintenance in an O_2_ carrier gas with 4% nitrous oxide. For lesion surgery, 3 µL of 6-OHDA (25 mM of 6-OHDA +0.025% ascorbic acid, Sigma Aldrich, St. Louis, MO, USA) was infused into the medial forebrain bundle (MFB) of the right hemisphere 1ul/min, using the following coordinates from bregma: −4 mm of the Anterior-Posterior axis (AP); −1.3 mm of the medial-lateral axis (ML); −7 mm below dura with the nose bar set at −4.5 [71,72]. For the transplantation surgery, VM pieces were dissected from E14 Wister rat embryos [73]. A cell suspension was prepared according to a standard protocol as described previously [74,75]. In brief, the dissected VM sections were washed 3 times with Dulbecco’s Modified Eagle Medium (DMEM) solution then incubated in a 1.5 mL of TryplE ^TM^ express solution and 30 mL of DNase solution at 37 °C for 20 min. The sections were then washed with DMEM/DNase solution (0.05% DNase) followed by a mechanical dissociation through trituration. After checking the viability, the cell suspension was centrifuged at 380 G for 3 min at room temperature and re-suspended in DMEM/DNase to prepare a final concentration of one third of a VM per 1 µL. Using a 23-gauge stainless steel cannula, 2 µL of the cells suspension was infused intra-striatal at one side of two depths at a rate of 1 µL/90 s using the coordinates: AP: −0.5 mm; ML: −3 mm; DV −5 mm and −4 mm, nose set bar at −4.5 mm. Finally, the wounds were sutured with vicryl 4-0 sutures and the rats received 30 mL Metacam (5 mg/mL) and 5 mL 0.9% saline subcutaneously.

### 4.4. Treatments

L-dopa (Sigma Aldrich: 12 mg/kg s.c. on alternate days) was given in combination with benserazide HCL (Sigma Aldrich 12 mg/kg). Administration commenced 5 weeks prior to cell transplantation and continued for 12 weeks post transplantation. Exendin-4 (Tocris Bioscience, Bristol, UK) was administered intra-peritoneally i.p. at a dose of 0.5 µg/kg twice daily [24] while liraglutide (Novo Nordisk, Victoza^®^, Bagsværd, Denmark) was given once daily at dose of 100 µg/kg [76], doses were consistent with previous studies showing neuroprotection. Both drugs were started one day prior to cell transplantation and continued for 12 weeks. In the lesion only group of set 1, the exendin-4 was started 3 weeks post lesion and continued for 16 weeks.

### 4.5. Motor and L-dopa Induced Dyskinesia Tests

All behaviour tests were conducted after a 48 h washout from all experimental drugs. For the drug induced rotations, the rats were injected with either methamphetamine HCl (2.5 mg/kg i.p.). They were then Immediately placed in automated rotometers (Rotorat) where the frequency and the direction of rotations were counted for 90 min [71,77]. For the stepping test, the rat’s body was supported by the researcher with weight resting through one forepaw as the animals was moved along 1m of bench in a forehand direction over 10 s. Steps were expressed as a percentage of the contralateral forelimb [78]. For the vibrissae test, the whiskers of the rats were brushed against a surface edge to stimulate a paw placing reflex on the surface. Data is expressed as the percentage of contralateral reflexes compared the ipsilateral paw [79]. For the cylinder test, rats were placed in a Perspex cylinder (height: 33.5 cm, diameter: 19 cm) and video recorded for 5 min or until 20 weight bearing touches had been performed [79]. All hand tests expressed the contralateral paw movements as a percentage of the ipsilateral paw.

L-dopa-induced rotations were evaluated immediately after L-dopa injection using automated rotometers to record rotational behaviour over 3 h sessions. L-dopa-induced dyskinesia (LID) were evaluated using AIMs scale as described previously [80,81]. In brief, the rats were observed for 3 h after L-dopa injection in 20 min intervals. The AIMs score was rated based on the duration and amplitude of abnormal movements in 4 categories: axial torsion, forelimb dyskinesia, hindlimb dystonia and orolingual movements. Then the total score was calculated by summation the multiplying of the duration and the amplitude score of each subtype.

### 4.6. Blood Sampling, Perfusion and Fixation

Nine hours prior to perfusion, food was removed from the cages and food hopper. Sodium pentobarbital (Euthatal, Merial, UK) 200 mg/mL administered i.p. to anesthetize the rats and blood collected via cardiac puncture from the left ventricle using coated syringes and transferred to EDTA containing tubes. Transcardial perfusion with 0.9% phosphate buffered saline was followed by 4% paraformaldehyde in 0.01 M phosphate buffer salt (PBS) solution. Brains were then extracted and post-fixed in 4% PFA solution for 4 h followed by immersion in a 25% sucrose solution. A freezing microtome was used to cut the brain into 30 μm sections in 1:12 series.

### 4.7. Plasma Sample Analysis

Blood samples were mixed with aprotinin solution in EDTA tubes and centrifuged to keep the supernatant at −80 °C. For glucose analysis, YSI 2300 STAT PLUS™ analyser was used to measure plasma glucose level. Two readings of glucose level (mmol/L) was given for each sample then the average value was considered. For Insulin analysis, luminex assay was performed using Rat Metabolic Magnetic Bead Panel from Millipore Company (RMHMAG-84k) and Bio-Plex 200 system powered by luminex x-map TM technology. A standard procedure from Milliplex map kit guidelines was followed to detect the plasma insulin concentration in pg/mL [82]. Insulin resistance index (Homeostatic Model Assessment of Insulin Resistance (HOMA-IR)) was calculated from the fasting level of glucose and insulin from the following equation: HOMA-IR = (Glucose in mmol/L) × (Insulin in pg/mL)/22.5) [83].

### 4.8. Immunohistochemistry

Proteins were visualised in free floating sections with 3,3′-Diaminobenzedine (DAB) or double immunofluorescence. Sections were blocked with 3% serum in triton/TBS solution (1.2% Tris Base and 0.9% NaCl) and then incubated with primary antibody overnight at room temperature followed by washing except IRS-1 pS 1011 which was incubated with 10% of horse serum and with the primary antibody at 4 °C. For DAB assays, sections were then incubated with biotinylated secondary antibody for 2 h (1:200, Vector Labs, Burlingame, CA, USA) followed by washing and incubation with avidin-biotin complex (1:200, Vectastatin ABC kit, Vector Labs), before incubation with 0.1% DAB. For double fluorescence staining, sections were incubated with fluorescent secondary antibody, cy3 (Jackson Immuno Research, 1:500, West Grove, PA, USA) or Alex flour 488 (Thermo Fisher Scientific, 1:500, Waltham, MA, USA), then the process repeated for the second primary antibody. All sections were mounted on gelatinized slides followed by dehydration with alcohol and delipidation with xylene followed by coverslipping with DPX. The primary antibodies used were rabbit anti-TH (Millipore, 1:1000, Burlington, MA, USA), mouse anti-CD45 (Ab Serotec, 1:500, Raleigh, NC, USA), mouse anti-CD11b (Ab Serotec, 1:2000), rabbit anti-GLP-1R (Abcam, 1:200, Cambridge, UK) and IRS-1 pS 1011 antibody (Cell Signalling, 1:100, Danvers, MA, USA).

### 4.9. Histological Analysis

For the analysis of dopaminergic neurons in the graft, 1:6 brain sections underwent TH IHC with DAB visualisation. TH+ cells were counted on 20× magnification bright field using a Leica light^®^ microscope. The number was corrected by applying the Abercrombie equation: N = ∑ {*n* × F × T/(T + H)} where N = Total corrected number, *n* = number of the counted cells, F = frequency of the sections, T = thickness of the sections (30 μm), and H = mean diameter of the cells [84]. The volume of dopaminergic neurons in the graft was calculated by summation of the area of TH+ graft in all the striatal sections multiplied by section thickness and series frequency. The graft area was measured from images captured at 4× magnification by using ImageJ (National Institute of Health, Bethesda, MD, USA).

The extent of the SN dopaminergic lesion was calculated at the level of medial terminal accessory nucleus of the optic tract where the SN is delineated from the vental tegmental area as a percentage of the number of TH+ cells in on the lesioned hemisphere as a percentage of the intact side.

For analysis of inflammation, CD45^+^ leukocytes were counted in the transplanted striatum under Feltiz dilux 22^®^ microscope in 1:12 sections, then the total number corrected with the Abercrombie equation. CD11b+ microglia were quantified by measuring optical density (OD) in the middle section of the graft within a 40 µm distance of the graft border. The OD was measured in corresponding places of the intact striatum with the cortex used as background control. OD around the graft was expressed as a relative percentage of the intact striatum after normalisation to background. Images were obtained at 4× magnification and analysed by ImageJ.

### 4.10. Immunocytochemistry

A suspension of E14 VM cells were plated and fixed with 4% PFA in 24-well poly-D-lysine cover-slipped plate. Cells were blocked with 3% serum plus 1% bovine albumin serum then incubated in the blocking solution with a primary antibody overnight at 4 °C. They were then washed and placed in a fluorescent secondary antibody cy3 (red) or Alex flour 488 (green). The same process was repeated to stain the second marker. Antibodies used were mouse anti-TH (Millipore, 1:1000); rabbit anti-GLP-1R (Abcam, 1:100); mouse BIII tubulin (Abcam, 1:50); and goat anti-SOX2 (Santacruz, 1:50, Santa Cruz, CA, USA).

### 4.11. Protein Lysis and Western Blot Analysis

Striatum, substantia nigra, frontal cortex and hippocampus were dissected from 2 adult female SD rats and stored on −80 °C. In addition, 7 E14 VM were collected from embryos of two Wistar rat dams and pooled, stored at −80 °C. The tissues were homogenized in tubes containing ceramic beads using the Precellys 24 homogenizer. They were mixed with a lysis buffer including anti-protease and anti-phosphatase followed by centrifugation (17,000× *g* for 60 min) to obtain the supernatant. Protein concentration estimation was followed using Bicinchoninc acid assay kit (Thermo Scientific Lab).

20–30 µg of proteins were loaded onto 10% SDS-PAGE running gel in an electrophoresis chamber at 200 V for 1–3 h. Then, the extracted proteins were transferred to a nitrocellulose blotting membrane using semi-dry membrane transfer inside electric field at 39 A for 1 h. Then, the blotting membrane washed and blocked with 5% skimmed milk in TBS tween solution incubation with primary antibody overnight at 4 °C. The membrane washed and placed in horseradish peroxidase conjugated secondary antibody solution followed by washing. Bands were developed using SuperSignal^®^ West Dura kit and visualised using gel imaging Syngene^®^ G BOX linked to GeneSys^®^ software (Daly City, CA, USA).

### 4.12. Statistical Analysis

All data was analysed using GraphPad Prism v8. Data were first examined for normal distribution using the Shapiro–Wilk test for normality. The non-normally distributed data were transformed to square root (sqrt) values before the analysis: TH^+^ cell count; CD45^+^ cell count; insulin level and HOM-IR value. The data pertaining to the motor tests were normalized and expressed as a percentage of the mean baseline time point (pre-transplantation or pre-exendin-4). Kruskal- Wallis test followed by Dunn’s post hoc test was performed for the non-parametric data of AIMS score. Two-way ANOVA was applied for the motor tests, L-dopa induced rotation test, histological and blood analysis data. Bonferroni’s post hoc test was used for pairwise comparisons and statistical adjustment for all data. Unpaired *t*-test was used to analyse the motor tests of lesion vs. lesion + Ex-4 groups; lesion vs. graft groups. In the amphetamine-induced rotation test, the (graft + Ex-4) and (graft + LD + lira) groups each had one outlier detected and excluded from the analysis. The outlier was considered if it has a value lower or higher than double the standard deviation from the data mean. For the assessments of graft function, the comparison between behavioural data of the lesion group and the graft plus saline group were analysed separately. Data are presented as mean ± standard error of the mean. Significance was considered at a *p* < 0.05.

## 5. Conclusions

Here we confirm that GLP-1R agonists may offer additional benefits to support the survival and function grafted cells for the treatment of Parkinson’s but that this may be in a L-dopa dependent manner. Importantly, co-administration of exendin-4 with L-dopa led to the development of markers indicating insulin resistance. Understanding the implications of this in depth will be important for the future use of GLP-1 agonists.

## Data Availability

Data is available on request.

## Supplementary Materials

The following are available online at https://www.mdpi.com/article/10.3390/ijms222212346/s1.
