# Supplementary material for: L-dopa-Dependent Effects of GLP-1R Agonists on the Survival of Dopaminergic Cells Transplanted into a Rat Model of Parkinson Disease
Source: Int J Mol Sci. 2021 Nov 16;22(22):12346. doi: 10.3390/ijms222212346 (PMC8618072; doi:10.3390/ijms222212346)
Supplement: Supplementary file 1 [file ijms-22-12346-s001.zip › ijms-1414421-supplementary/Supplementary figure_S1 .pdf]

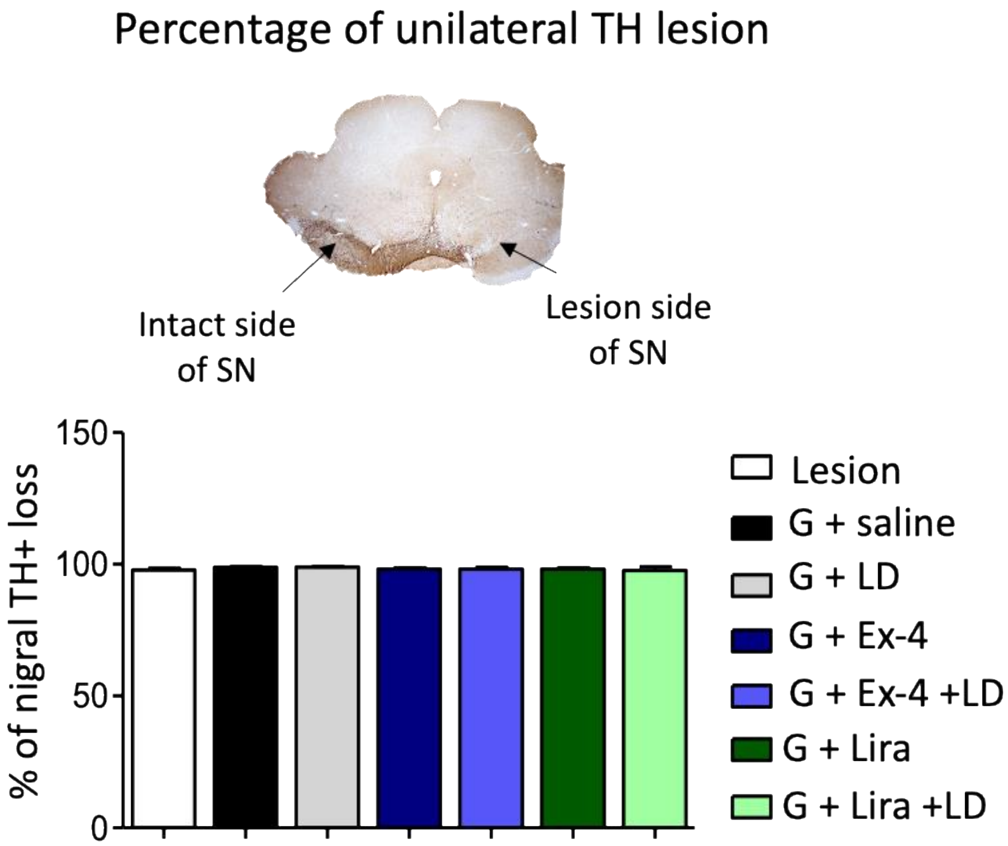

Supplementary figure S1. percentage of TH+ cells loss in the lesioned substantia nigra: more than 97% of nigral dopaminergic neurons was degenerated in the right hemisphere (lesioned side) compared to the intact side of all groups. SN= substantia nigra, G= graft; LD = L-dopa, Ex-4 = Exendin-4; Lira = Liraglutide.
