# Supplementary material for: L-dopa-Dependent Effects of GLP-1R Agonists on the Survival of Dopaminergic Cells Transplanted into a Rat Model of Parkinson Disease
Source: Int J Mol Sci. 2021 Nov 16;22(22):12346. doi: 10.3390/ijms222212346 (PMC8618072; doi:10.3390/ijms222212346)
Supplement: Supplementary file 1 [file ijms-22-12346-s001.zip › ijms-1414421-supplementary/Supplementary figure_S2.pdf]

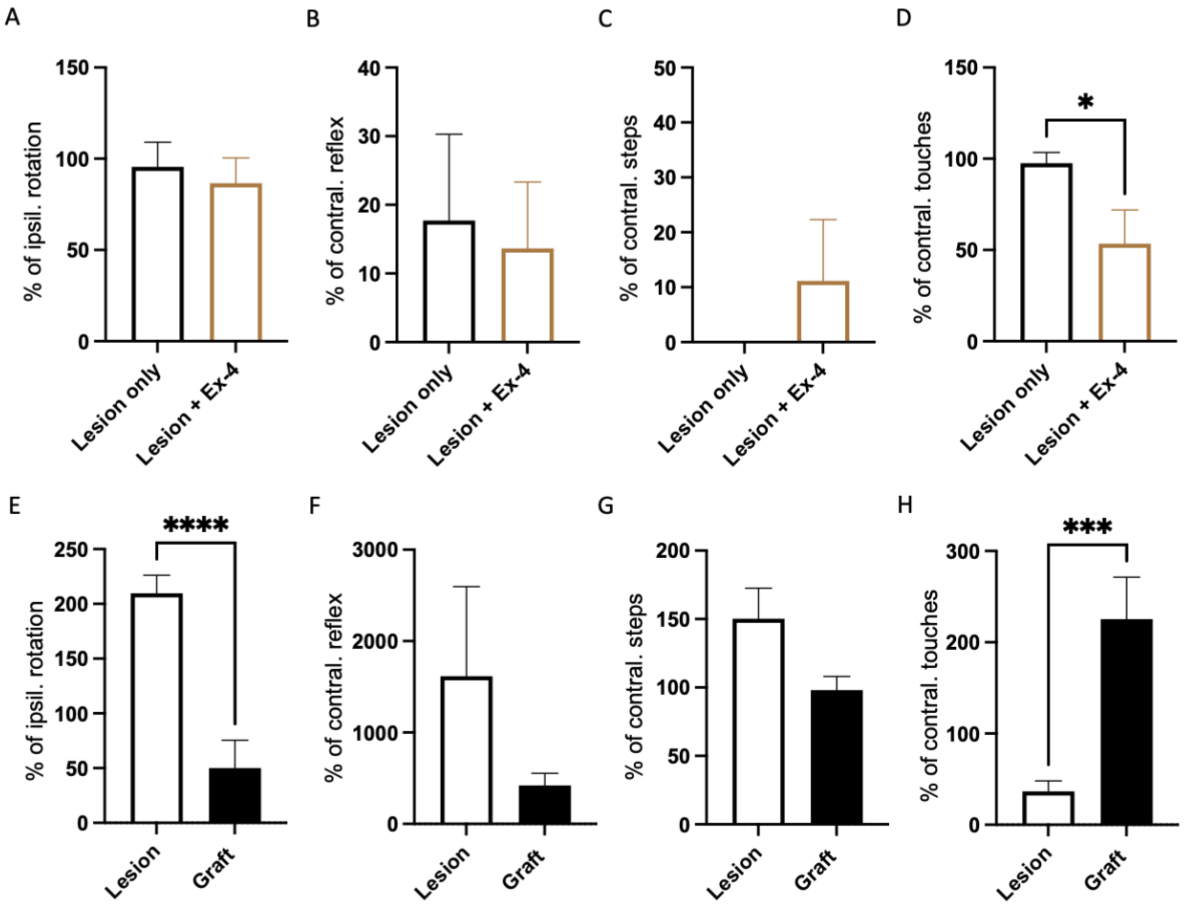

Supplementary figure S2. effect of exendin-4 and E14 VM cells graft on the motor and behavioural tests of the long term 6-OHDA complete lesion rat PD model. (A-D) The comparison between the lesion and lesion + Ex-4 groups at (A) amphetamine induced rotation test, (B) vibrasae test, (C) stepping test and (D) cylinder test on week 16 of exendin-4 treatment. The data were expressed as a percentage of the baseline (pre-exendin-4 treatment) in number of ipsilateral rotations, contralateral reflex, contralateral steps and contralateral touches, respectively (E-H) The comparison between lesion and graft groups at (E) amphetamine induced rotation test, (F) vibrasae test, (G) stepping test and (H) cylinder test on week 12 post-transplantation. The data were expressed as a percentage of the baseline (pre-transplantation time point) in number of ipsilateral rotations, contralateral reflex, contralateral steps and contralateral touches, respectively. n= [(A-D), lesion (8), lesion + Ex-4 (8); (E-H) lesion (9) graft (7)]. t- test, *p< 0.05, ***p< 0.001. Ex-4 = exendin-4; n.s. = not significant.
