# Supplementary material for: L-dopa-Dependent Effects of GLP-1R Agonists on the Survival of Dopaminergic Cells Transplanted into a Rat Model of Parkinson Disease
Source: Int J Mol Sci. 2021 Nov 16;22(22):12346. doi: 10.3390/ijms222212346 (PMC8618072; doi:10.3390/ijms222212346)
Supplement: Supplementary file 1 [file ijms-22-12346-s001.zip › ijms-1414421-supplementary/Supplementary figure_S3.pdf]

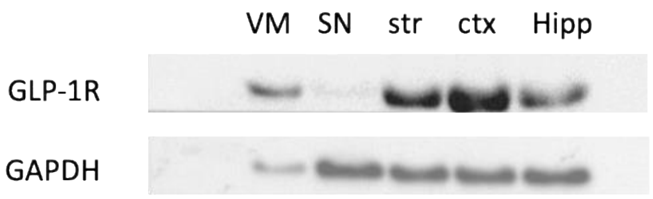

Supplementary figure S3. Western Blot analysis illustrated detection of GLP-1R protein (size 53 kDa) in E14 VM sections of Wistar rat embryos and also in Substantia nigra (SN), striatum (str), frontal cortex (ctx) and hippocampus (Hipp) of SD female adult rat; GAPDH was used as a house keeper (size 37 kDa).
